# CBESW: Sequence Alignment on the Playstation 3

**DOI:** 10.1186/1471-2105-9-377

**Published:** 2008-09-17

**Authors:** Adrianto Wirawan, Chee Keong Kwoh, Nim Tri Hieu, Bertil Schmidt

**Affiliations:** 1 School of Computer Engineering, Nanyang Technological University, Singapore

## Abstract

**Background:**

The exponential growth of available biological data has caused bioinformatics to be rapidly moving towards a data-intensive, computational science. As a result, the computational power needed by bioinformatics applications is growing exponentially as well. The recent emergence of accelerator technologies has made it possible to achieve an excellent improvement in execution time for many bioinformatics applications, compared to current general-purpose platforms. In this paper, we demonstrate how the PlayStation^® ^3, powered by the Cell Broadband Engine, can be used as a computational platform to accelerate the Smith-Waterman algorithm.

**Results:**

For large datasets, our implementation on the PlayStation^® ^3 provides a significant improvement in running time compared to other implementations such as SSEARCH, Striped Smith-Waterman and CUDA. Our implementation achieves a peak performance of up to 3,646 MCUPS.

**Conclusion:**

The results from our experiments demonstrate that the PlayStation^® ^3 console can be used as an efficient low cost computational platform for high performance sequence alignment applications.

## Background

Sequence alignment is a popular bioinformatics application that determines the degree of similarity between nucleotide or amino acid sequences which is assumed to have same ancestral relationships. The optimal local alignment of a pair of sequences can be computed by the dynamic programming (DP) based Smith-Waterman (SW) algorithm[1]. However, this approach is expensive in terms of time and memory cost. Furthermore, the exponential growth of available biological data[2] means that the computational power needed is growing exponentially as well.

The recent emergence of accelerator technologies such as FPGAs, GPUs and specialized processors have made it possible to achieve an excellent improvement in execution time for many bioinformatics applications, compared to current general-purpose platforms. However, special-purpose hardware implementations such as FPGAs [3,4] tend to be very expensive and hard-to-program. Hence, they are not suitable for many users. Recent usage of easily accessible accelerator technologies to improve the search time of the SW algorithm include Intel SSE2[5], GPU[6] and CUDA[7].

Farrar[5] exploits the SSE2 SIMD multimedia extension of general-purpose CPUs. His implementation utilizes vector registers, which are parallel to the query sequence and are accessed in a striped pattern. Similar to the implementation by Rognes [8], a query profile is calculated only once for each database search. However, Farrar's implementation allows moving the conditional calculation of the *F*-matrix outside the inner loop. Therefore, this implementation achieves a speed up of factor 2–8 over the previous SIMD implementations by Wozniak[9] and Rognes[8].

Liu et al. [10] first reported the implementation of the Smith-Waterman algorithm on graphics hardware. The SW algorithm is implemented using the streaming architecture of GPUs by reformulating it in terms of computer graphics primitives. The implementation relies on OpenGL, in which a conversion of the problem to the graphical domain is needed, as well as a reverse procedure to convert back the results. Although, it achieves a high efficiency, programming in OpenGL requires specialized skills. Therefore, Manavski[7] re-implemented the SW algorithm on a GPU with the recently released C-based CUDA programming environment. The implementation performs from 2 to 30 times faster than any other previous attempt available on commodity hardware.

In this paper, we demonstrate how the *PlayStation*^® ^3 (PS3), a commodity hardware powered by the Cell Broadband Engine[11], can be used as a low cost computational platform to accelerate the Smith-Waterman algorithm. Our implementation is able to outperform both the striped method on an Intel Core 2 Duo as well as the CUDA-based GPU implementation on a GeForce 8800 GTX.

### The Smith-Waterman Algorithm

The Smith-Waterman algorithm is used to determine the optimal local alignment between two nucleotide or protein sequences. The algorithm compares two sequences by computing the similarity score by means of dynamic programming (DP). Two elementary operations are used: substitution and insertion/deletion (also called a gap operation). The original algorithm was proposed by Smith and Waterman[1] with a complexity of O(m^2^n) and was improved by Gotoh[12] to run at O(mn).

Consider two strings *S*1 and *S*2 with length *m *and *n*, respectively. The Smith-Waterman algorithm computes the similarity value *M*(*i*, *j*) of two sequences ending at position *i *and *j *of the two sequences *S*1 and *S*2, respectively. For affine gap penalties, i.e. *α *≠ *β*, the computation of *M*(*i*, *j*), for 1 ≤ *i *≤ *m*, 1 ≤ *j *≤ *n*, is given in the following equations 1–3:

(1) *M*(*i*, *j*) = max{*M*(*i *- 1, *j *- 1) + *sbt*(*S*1[*i*]), *S*2[*j*], *E*(*i*, *j*), *F*(*i*, *j*), 0},

(2) *E*(*i*, *j*) = max{*M*(*i*, *j *- 1) - *α*, *E*(*i*, *j*-1) - *β*},

(3) *F*(*i*, *j*) = max{*M*(*i *- 1, *j*) - *α*, *F*(*i *- 1, *j*) - *β*},

where *sbt *is a character substitution cost table, *α *is the cost of the initial gap; *β *is the cost of the following gaps. For linear gap penalties, i.e. *α *= *β*, the above recurrence relations can be simplified as shown in equations 4:

(4) *M*(*i*, *j*) = max{*M*(*i *- 1, *j *- 1) + *sbt*(*S*1[*i*]), *S*2[*j*], *M*(*i*, *j *- 1) - *α*, *M*(*i *- 1, *j*) - *α*}

Initialization values are given as the following: for 0 ≤ *i *≤ *m*, 0 ≤ *j *≤ *n*, *M*(*i*, 0) = *M*(0, *j*) = *E*(*i*, 0) = *F*(*0*, *j*) = 0. Each position of the matrix *M *is a similarity value. The two segments of *S*1 and *S*2 producing this value can be determined by a trace-back procedure.

**Figure 1:**
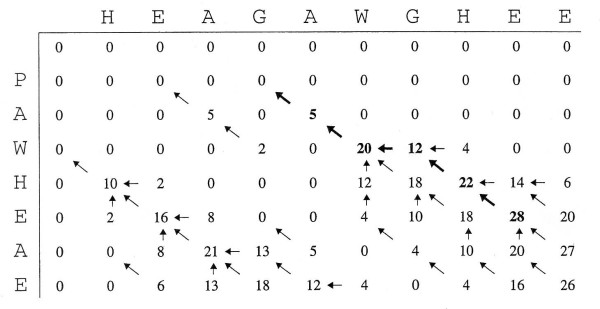
**Sequence alignment of YPKIEAIY and MPKIIEAIYEN**. An example of computing the local alignment between two sequences PAWHEAE and HEAGAWGHEE using the Smith-Waterman algorithm with the BLOSUM 50 scoring matrix[13]. The highest score in the matrix (+28) is the optimal score for the alignment. The trace-back procedure, shown in form of arrows, shows that the optimal local alignment is AW-HE and AWGHE.

Figure 1 illustrates an example of computing the local alignment between two sequences PAWHEAE and HEAGAWGHEE using the Smith-Waterman algorithm with the BLOSUM 50 scoring matrix [13]. The highest score in the matrix (+28) is the optimal score for the alignment. The trace-back procedure, shown in form of arrows, shows that the optimal local alignment is AW- HE and AWGHE.

### Cell Broadband Engine Architecture

The Cell Broadband Engine[14] (Cell BE) is a recently introduced single-chip heterogeneous multi-core processor, which is developed by Sony, Toshiba and IBM. The Cell BE offers a unique assembly of thread-level and data-level parallelization options. It is operating at the upper range of existing processor frequencies (3.2 GHz for current models) and is projected to run at more than 5 GHz in the near future. Several examples of bioinformatics applications that has been ported to the Cell BE architecture include Folding@Home[15], FASTA[16], ClustalW[16] and RAxML[17].

**Figure 2:**
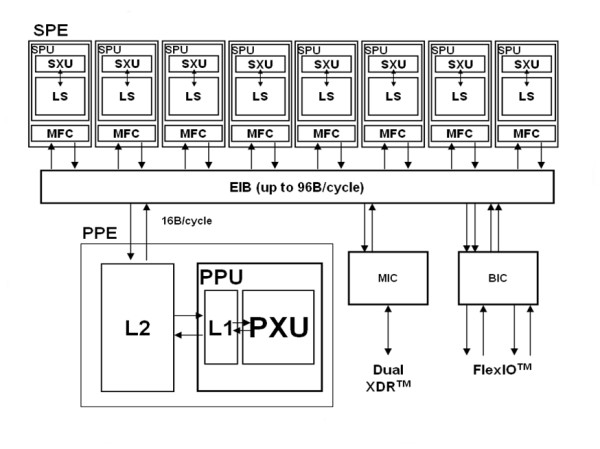
**Block diagram of the Cell BE Architecture**. The Cell BE architecture consists of 1 PPE and 8 SPEs. However, in the Play Station^® ^3, only 6 SPEs are available.

The Cell BE combines an IBM PowerPC Processor Element (PPE) and eight Synergistic Processor Elements (SPEs)[11]. An integrated high-bandwidth bus called the Element Interconnect Bus (EIB) connects the processors and their ports to external memory and I/O devices. The block diagram of the Cell BE architecture is shown in Figure 2.

The PPE is a 64-bit Power Architecture core and contains a 64-bit general purpose register set (GPR), a 64-bit floating point register set (FPR), and a 128-bit Altivec register set. It is fully compliant with the 64-bit Power Architecture specification and can run 32-bit and 64-bit operating systems and applications. Each SPE is able to run its own individual application programs. Each SPE consists of a processor designed for streaming workloads, a local memory, and a globally coherent Direct Memory Access (DMA) engine. The EIB is a 4-ring structure, and can transmit 96 bytes per cycle, for a bandwidth of 204.8 Gigabytes/second. The EIB can support more than 100 outstanding DMA requests.

The most distinguishing feature of the Cell BE lies within the variety of the processors it has, i.e. the PPE and the SPEs. Heterogenous multi-core systems can lead to decreased performance if both the operating system and application are unaware of the heterogeneity. The PPE is designed to run the operating system and, in many cases, the top-level control thread of an application, while the SPEs is optimized for compute intensive applications, hence, providing the bulk of the application performance.

The SPE can access RAM through direct memory access (DMA) requests. The DMA transfers are handled by the Memory Flow Controller (MFC). The MFC provides the interface, by means of the EIB, between the local storage of the SPE and main memory. The MFC supports DMA transfers as well as mailbox and signal-notification messaging between the SPE and the PPE and other devices. Data transferred between local storage and main memory must be 128-bit aligned. The size of each DMA transfer can be at most 16 KB. DMA-lists can be used for transferring large amounts of data (more than 16 KB). A list can have up to 2,048 DMA requests, each for up to 16 KB.

The PS3 uses the Cell Broadband Engine as its CPU, hence making it possible for users to create a high-powered computing environment for a fraction of the cost of a Cell Blade server. The PS3 utilizes seven of the eight SPEs, in which the eighth SPE is disabled to improve chip yields, i.e. chips do not have to be discarded if one of the SPEs is defective. Only six of the seven SPEs are accessible to developers as one is reserved by the operating system. The power requirement for the PS3 is 120 V AC, 60 Hz and the power consumption approximately 380 W. Generally available PS3's can be used for scientific high performance computing through installation of Linux (e.g. Red Hat or Yellow Dog). Programs can be developed the using freely available C-based Cell BE SDK [18]. At the time of this writing, the retail price of the PlayStation^® ^3 is US$ 399 for 40 GB and US$480 for 60 GB, while the retail price of the Nvidia GeForce 8800GTX card is US$529, and a Dell Optiplex 745 with Intel Core 2 Duo 2.4 GHz processor is US$871. A QS20 Blade Server with two Cell BE chips has a retail price of US$18,995. Thus, the PS3 offers a good alternative to other accelerator technologies.

## Methods

### Cell BE Mapping

Our sequence alignment implementation [see Additional file 1, 2 and 3] uses affine gap penalties and utilizes the 128-bit wide SIMD vector registers of the SPEs for optimization. The vectorization strategy is based on a column-based approach[5,8]. It also employs a static load balancing strategy, which means that the work load is known at the start and distributed equally across the SPEs. The code is written in C together with the Cell BE SIMD Multimedia Extension Language intrinsics and SPU intrinsics for portability. DMA transfers and mailbox functions are used for communication purposes.

A list of SPU Low-Level Specific and Generic Intrinsics used in our vectorized implementation, divided into categories, is shown in Table 1. Constant Formation Intrinsics, Arithmetic Intrinsics, Compare, Branch and Halt Intrinsics, Bits and Mask Intrinsics, Logical Intrinsics, Shift and Rotate Intrinsics and Scalar Intrinsics have been employed to access hardware features, which are not easily accessible from a high level language in order to obtain the best performance from the Cell BE.

**Table 1:** List of SPU Low-Level Specific and Generic Intrinsics used in the implementation

**Category of Intrinsics**	**SPU Low-Level Specific and Generic Intrinsics used**
Constant Formation Intrinsics.	spu_splats
Arithmetic Intrinsics	spu_add spu_sub
Compare, Branch and Halt Intrinsics	spu_cmpgt
Bits and Mask Intrinsics	spu_sel
Logical Intrinsics	spu_or spu_and spu_nor spu_nand
Shift and Rotate Intrinsics	spu_slqwbyte spu_rlmaskqwbyte spu_rlmaska
Scalar Intrinsics	spu_extract

List of SPU Low-Level Specific and Generic Intrinsics used in the implementation are listed and sorted into categories, as shown below. More details about the syntax and semantics of these Intrinsics can be found in [19].

**Figure 3:**
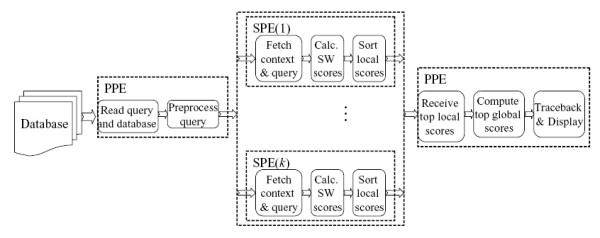
**Mapping of the different stages of database scanning with SW onto the Cell B.E**. The block diagram shows the mapping of the different stages of database scanning with SW onto the Cell BE.

**Figure 4:**
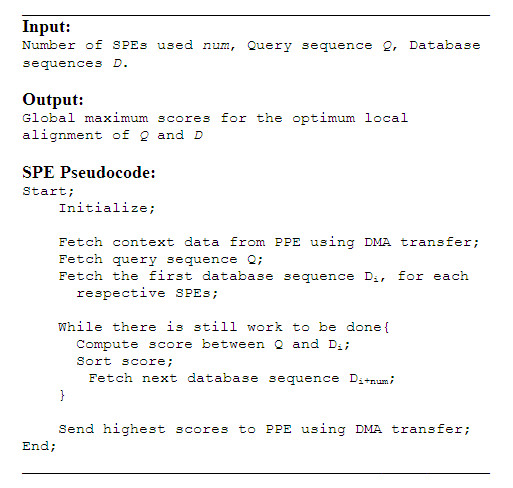
**Pseudocode of the Cell BE mapping**. Pseudocode of the SPE code for the Cell BE mapping.

Figure 3 illustrates the mapping of different stages of SW-based protein sequence database scanning application onto the Cell BE. The PPE starts by reading the query and the database from the respective files and then pre-processes the query sequences such that they are suitable for vector operations. The pre-processed query sequence, together with some context data, is sent to each respective SPEs, which in turn will generate its own query profile. This process is done using DMA transfers, namely *mfc_get *and *mfc_put*. Given a database *D *consisting of |*D*| sequences and *k *SPEs. Each SPE aligns the query sequence to $\frac{\left|D\right|}{k}$ database sequences. Pseudocode of the mapping is illustrated in Figure 4. Scores obtained from those alignments are sorted locally in the SPEs and the *b *highest scores are sent to the PPE, where they are sorted once again to obtain the *b *overall highest scores.

Due to the fact that the SPEs only have 256 Kbytes of local memory, which have to store program code and data, memory allocation is crucial for the SPE. The current longest sequence in the Swiss-Prot database is 35,213 amino acids (accession number A2ASS6). In order to accommodate for longer protein sequence in the future, we allocate dynamic memory for the database sequences of up to 64,000 amino acids per sequence. Due to these limitations, the maximum query sequence length allowed for our implementation is limited to 852.

### Query Profile

In order to calculate *M*(*i*, *j*) in the SW DP matrix, the value *sbt*(*S*_1_[*i*], *S*_2_[*j*]) needs to be added to *M*(*i*-1, *j*-1). To avoid performing this table lookup for each element in the DP matrix, Rognes[8] and Farrar [5] suggested calculating a *query profile *parallel to the query sequence beforehand.

Assuming that *S*_1_, *S*_2 _∈ Σ* and *S*_1 _is the query sequence, the query profile is defined as a set *P *= {*P*_*x *_| *x*∈Σ} consisting of |Σ| numerical strings of length *l*_1 _each, where *l*_1 _= |*S*_1_|. Each string *P*_*x *_∈ *P *consists of all substitution table values that are needed to compute a complete column *j *of the DP matrix for which *S*_2_[*j*] = *x*. Pre-computing the query profile greatly reduces the amount of substitution table lookup in the SW DP matrix computation, since |Σ| is usually much smaller than |*S*_2_|.

**Figure 5:**
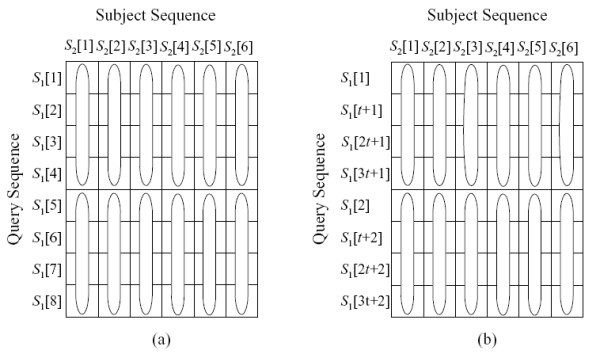
**The query profile layout**. The query profile layout for (a) sequential method, (b) striped method.

The query profile can be calculated in a straightforward *sequential layout *[8] or in a more complex *striped layout *[5], as shown in figure 5. The values in the query profile for sequential and striped layout are defined in equation 4 and 5, respectively:

(4a) *P*_*x*_[*i*] = *sbt*(*S*_1_[*i*], *x*), for all 1 ≤ *i *≤ *l*_1_,

(5) $$\begin{array}{cc} P_{x}[i]=sbt\left(S_{1}\left[\left(\left(\left(i-1\right)\%p\right)t\right)+\left\lfloor \frac{i-1}{p}\right\rfloor +1\right],x\right) & \text{for all }1\leq i\leq l_{1} \end{array}$$

where *p *is the number of segments and *t *is the segment length.

In the striped layout, *p *corresponds to the number of elements that can be processed in a SIMD vector register (e.g. for 128-bit wide SIMD registers, *p *= 8 when using 16-bit precision). The length of each segment, *t *is defined in equation 6.

(6) *t *= ⌈(*l*_1 _+ *p *- 1)/*p*⌉

Both approaches allow efficient vectorization on SSE2-compatible processors using the corresponding SIMD instruction set. Using the pre-calculated query profile, the computation of the DP matrix can be performed in column-wise order. Due to the simplified dependency relationship and parallel loading of the vector scores from memory, fast DP matrix calculations can be achieved. The advantage of the striped layout compared to the sequential layout is that data dependencies between vector registers are moved outside the inner loop. For instance, when calculating vectors for the DP matrices *H *or *F *with the sequential layout, the last element in the previous vector has to be moved to the first element in the current vector. When using the striped query layout, this needs to be done just once in the outer loop when processing the next subject sequence character.

### Saturation Arithmetic

The inner loop of the algorithm requires saturation arithmetic, namely saturated additions and saturated subtractions. The Cell BE lacks the saturation arithmetic support, leaving the tasks to be handled by software instead of direct hardware support. In order to tackle this problem, we introduced two new functions, namely *spu_adds *and *spu_subs *to handle saturated additions and saturated subtractions, respectively.

## Results and discussion

In this section, we analyze the performance of our parallel algorithm for various query sequence lengths using sequences from Swiss-Prot database. Searches for 18 query sequences with various lengths between 63 to 852 amino acids were performed. The accession numbers of the query sequences used are O29181, P03630, P02232, P05013, P14942, P00762, P53675, Q8ZGB4, P10318, P07327, P01008, P10635, P58229, P25705, P42357, P21177, Q38941 and O60341, respectively. All queries were run against Swiss-Prot release 55.2 comprising 130,497,792 amino acids in 362,782 sequence entries. The gap-open penalty used was 10 and the gap-extension penalty used was 2. The scoring matrix used in the testing was BLOSUM45. All experiments were carried out on a standalone PlayStation^® ^3 machine, with Yellow Dog Linux 5.0 operating system and the Cell Software Development Kit (SDK) 2.0.

The performance statistics measured are then converted to the following measurements, i.e. computational time and Mega Cell Updates Per Second (MCUPS). Given a query sequence of size Q and a database of size D, the MCUPS rating (million cell updates per second) is calculated by Equation 7.

(7) $$\frac{\left|Q\right|\times \left|D\right|\times 10^{6}}{t}$$

where

|*Q*| = size of query sequence in amino acids

|*D*| = size of database sequences in amino acids

*t *= run time (including input from file, initialization and result output)

Table 2 shows the performance of our parallel algorithm on the above mentioned datasets. By using 6 SPEs available in the PS3, our parallel algorithm reaches a peak performance of 3,646.48 MCUPS for a query sequence of length 852 (accession number O60341).

**Table 2:** Performance evaluation

**Accession number**	**Query Sequence Length**	**CBESW (seconds)**	**CBESW (MCUPS)**
O29181	63	18.45	445
P03630	127	19.05	869
P02232	143	19.17	973
P05013	189	19.6	1,258
P14942	222	20.12	1,439
P00762	246	20.24	1,586
P53765	255	20.43	1,628
Q8ZGB4	361	22.04	2,137
P10318	362	22.06	2,141
P07327	374	22.39	2,179
P01008	464	23.18	2,612
P10635	497	23.69	2,737
P58229	511	24.43	2,729
P25705	553	24.74	2,916
P42357	657	26.64	3,218
P21177	729	28.06	3,390
Q38941	850	30.45	3,642
O60341	852	30.49	3,646

Performance evaluation of our implementation, in terms of computational time and MCUPS. All queries were run against Swiss-Prot release 55.2 comprising 130,497,792 amino acids in 362,782 sequence entries. Eighteen query sequences of length 63 to 852 amino acids were used. The gap-open penalty used is 10 and the gap-extension penalty used was 2. The BLOSUM45 scoring matrix was used.

We have compared the performance of our CBESW implementation with other publicly available implementations of SW-based protein database scanning, namely SSEARCH[20], Striped Smith-Waterman[5] and CUDA[7]. The query sequences, as well as their respective Swiss Prot accession numbers, used in the different performance comparisons are shown in Table 3.

**Table 3:** List of query sequences used in different performance comparisons

**Accession number**	**Query Sequence Length**	**Comparison w/SSEARCH**	**Comparison w/Striped SW**	**Comparison w/CUDA**
O29181	63	√	√	√
P03630	127	√	√	√
P02232	143			√
P05013	189			√
P14942	222			√
P00762	246			√
P53765	255	√	√	√
Q8ZGB4	361	√	√	√
P10318	362			√
P07327	374			√
P01008	464			√
P10635	497			√
P58229	511	√	√	
P25705	553			√
P42357	657	√	√	√
P21177	729	√	√	√
Q38941	850	√	√	√
O60341	852	√	√	√

List of query sequences, as well as their respective accession number, used in different performance comparisons with other publicly available implementations of SW-based protein database scanning, namely SSEARCH, Striped Smith-Waterman and CUDA.

**Figure 6:**
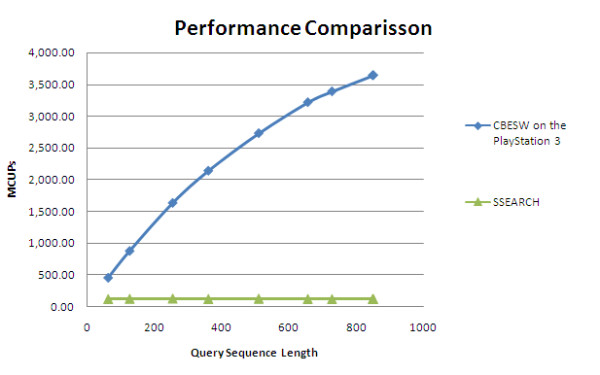
**Performance comparison with the SSEARCH implementation**. Performance comparison between our CBESW implementation with SSEARCH, in terms of MCUPS. All queries were run against Swiss-Prot release 55.2. Nine query sequences with lengths of 63 to 852 amino acids were used.

SSEARCH[20] is a SW implementation which is part of the FASTA[21] package. The SSEARCH performance is benchmarked on an Intel Core 2 Duo 2.4 GHz CPU with 1 GB RAM. Both execution cores were used in the experiment. As shown in Figure 6, for a query sequence of length 852 (accession number O60341), SSEARCH achieves a performance of 121.91 MCUPS. Thus, our implementation is over 30 times faster.

**Figure 7:**
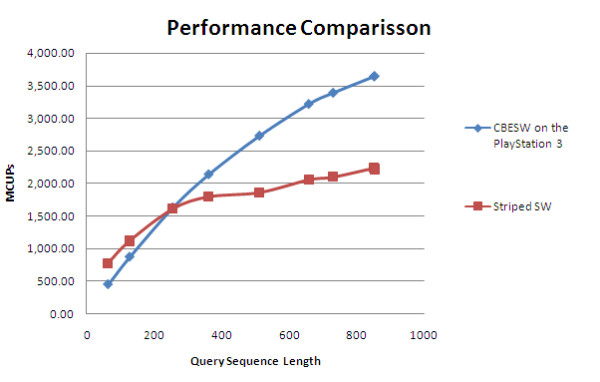
**Performance comparison with the Striped Smith-Waterman implementation**. Performance comparison between our CBESW implementation with Striped Smith-Waterman, in terms of MCUPS. All queries were run against Swiss-Prot release 55.2. Nine query sequences with lengths of 63 to 852 amino acids were used.

Figure 7 shows the performance comparison between PS3 and striped SW. Striped SW is also benchmarked on an Intel Core 2 Duo 2.4 GHz CPU with 1 GB RAM. Both execution cores were used in the experiment. As can be seen from the figure, for query sequences with length > 255 amino acids, our PS3 implementation achieves a higher MCUPS performance compared to striped SW. The PS3 peak performance is 1.64 times faster than striped SW for the query sequence of length 852.

**Figure 8:**
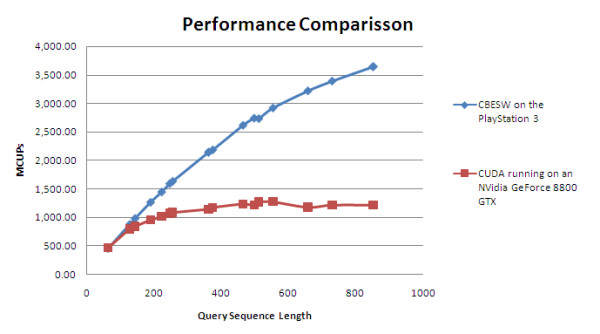
**Performance comparison with the CUDA implementation on a single Nvidia GeForce 8800GTX**. Performance comparison between our CBESW implementation with CUDA implementation on a single Nvidia GeForce 8800GTX, in terms of MCUPS. All queries were run against Swiss-Prot release 55.2. Seventeen query sequences with lengths of 63 to 852 amino acids were used. The scoring matrix used for the CUDA implementation was BLOSUM 50.

The performance comparison between the PS3 implementation and CUDA-SW on one Nvidia GeForce 8800GTX is shown in Figure 8. The CUDA implementation experiment was conducted with a GeForce 8800GTX 512 MB installed in a PC with a Dual-Core AMD Opteron 2210 1.8 GHz CPU, 2 GB RAM running Fedora 6. The substitution matrix used is BLOSUM50. As can be seen from the figure, our implementation achieves a better MCUPS performance. The PS3 peak performance is 3 times faster compared to the peak performance CUDA implementation on a single Nvidia GeForce 8800GTX.

## Conclusion

In this paper, we have demonstrated that the PlayStation^® ^3, powered by the Cell Broadband Engine, can be effectively used to accelerate a biological sequence alignment application. In order to derive an efficient mapping onto this type of heterogeneous multi-core architecture, we have utilized SIMD vectorization and parallel data partitioning and communication techniques.

Our implementation achieves a peak performance of 3,646.48 MCUPS for a query sequence of length 852. Hence, the peak performance of our implementation is 30.1 times and 1.64 times faster than SSEARCH and striped SW, on an Intel Core 2 Duo 2.4 GHz. The PS3 peak performance is also 3 times faster compared to the peak performance CUDA implementation on a single Nvidia GeForce 8800GTX.

The very rapid growth of biological sequence databases demands even more powerful high-performance solutions in the near future. Hence, our results are especially encouraging since high performance computer architectures are developing towards heterogeneous multi-core systems.

Due to the 256 KB memory limitation of the SPE local store, the maximum query sequence length in our current implementation is 852. One of the limiting factors is that the size of the query profile grows with the length of the query sequence. Part of our future work is therefore to tackle this limitation. A promising approach is to align subject sequences against separate chunks of the query profile. The complete query profile only needs to be stored once in the main memory instead of the local store of the SPE. This frees up more memory space for the SPEs and thus allows longer query sequences. Given a query sequence of length *l*, the query profile can be divided into *n *chunks in which each chunks contains a query profile of size *l*/*n*. The respective SPEs can then align a part of the chunk of the query profile it has and get the next chunk from outside memory via concurrent DMA transfer.

## Availability and requirements

▪ Project name: CBESW

▪ Project homepage: 

▪ Operating system(s): only tested with PlayStation^® ^3 with Yellow Dog Linux 5.0

▪ Programming language: C

▪ Other requirements: Cell SDK 2.0

▪ License: none

▪ Any restrictions to use by non-academics: none

## Abbreviations

CPU: Central Processing Unit; CUDA: Compute Unified Device Architecture; CUPS: Cells Updates Per Seconds; DP: Dynamic Programming; DMA: Direct Memory Access; FPGA: Field-Programmable Gate Arrays; GPU: Graphics Processing Unit; MFC: Memory Flow Controller; PPE: PowerPC Processor Element; SIMD: Single Instructions Multiple Data; SPE: Synergetic Processor Element; SSE: Streaming SIMD Extensions; SW: Smith-Waterman

## Competing interests

The authors declare that they have no competing interests.

## Authors' contributions

AW conceived the study, participated in its design, implementation and coordination, performed benchmark test cases and drafted the manuscript. TNH participated in the design and implementation of the source code. KCK participated in the creation of the manuscript. BS conceived the study, participated in the analysis and interpretation of data. All authors have read and approve the final manuscript.

## Supplementary Material

Additional file 1 The file is the compressed source code for the CBESW. Click here for file

Additional file 2 The file is the executable file for CBESW. The command to run it is ./CBESW. Click here for file

Additional file 3 Readme file for CBESW file. Click here for file
