# Supplementary material for: CBESW: Sequence Alignment on the Playstation 3
Source: BMC Bioinformatics. 2008 Sep 17;9:377. doi: 10.1186/1471-2105-9-377 (PMC2571991; doi:10.1186/1471-2105-9-377)
Supplement: Additional file 3 — Readme file for CBESW file. [file 1471-2105-9-377-S3.docx]

# Setting up CBESW Project Environment

## 1.1 Cell Box setup

### 1.1.1 Yellow Dog Linux operating system

*Prepare Yellow Dog Linux:*

1. Attach your PS3 to a hi-definition TV or monitor using an HDMI (digital) or Component (5 RCA jacks) cable. Composite (yellow RCA jack) is not supported as the resolution is too low.
2. Attach the game controller, a USB keyboard and mouse to your PS3. The game controller will be used to conduct all GameOS functions. The USB keyboard and mouse will be used once you enter the YDL installer.
3. Power on your PS3. If this is the first time you have used your PS3, you will be prompted to Select a language, Select a time zone, Set the time & date, and Set the username (more easily done with a USB keyboard).
4. Select: Settings ==> System Settings ==> Format Utility ==> Format Hard Disk ==> Yes ==> Yes
5. Then select a partition setting for the hard disk: Custom
6. You are presented with option to "Allot 10GB to the Other OS" or "Allot 10GB to the PS3 System." Allocating the majority for YDL (Other OS) is recommended, independent of the size of your drive.
7. Select Quick Format and "Yes". Your PS3 will now format its internal drive.
8. Press "X" on the controller to EXIT which will cause your PS3 to reboot.

*Install Yellow Dog Linux:*

1. Insert YDL Install DVD.
2. From the GameOS menu, select: Settings ==> System Settings ==> Install Other OS
3. Your PS3 will now scan the DVD for the bootloader installer and bootloader.
4. Confirm discovery of "/ps3/otheros/otheros.bld".
5. Press "X" on the controller to start the installation. The PS3 screen blanks while pulling files from the DVD.
6. You must now select "Other OS" in order to install YDL: Settings ==> System Settings ==> Default System ==> Other OS
7. When prompted to boot Other OS, select "Yes".
8. Under *kboot*: enter Anaconda
9. Follow the instruction on the screen to perform standard Linux installation: keyboard, language settings, drive partitioning, dependency check and package selection. (Note: Similar to Fedora Core Linux, Yellow Dog Linux is an *rpm*-based operating system)
10. Reboot the PS3. Under the bootloader, press ENTER. Now you can work with a Linux operating system on the PS3.

### 1.1.2 Cell Standard Development Kit (Cell SDK)

It is crucial to note that IBM’s Cell SDK is not designed to work on PS3 machine, i.e. there is no guarantee that the libraries can work properly on the Cell Box. However, by manual try and error process, a configuration of Cell SDK appears to be working on this Cell Box.

*Prepare Cell SDK v2.0:*

1. By default, Cell SDK v1.1 is installed. This has to be removed using Package Manager. To open package manager, select: Applications ==> Applications ==> Accessories ==> System Tools ==> Software Management ==> Add/Remove Software.
2. Select: Base System, uncheck “Cell Libraries”
3. Click “Apply”
4. Download the rpm packages for Cell SDK v2.0 into a folder, such as sdk2.0-20061129. The list of these rpm packages is shown in Figure 3.


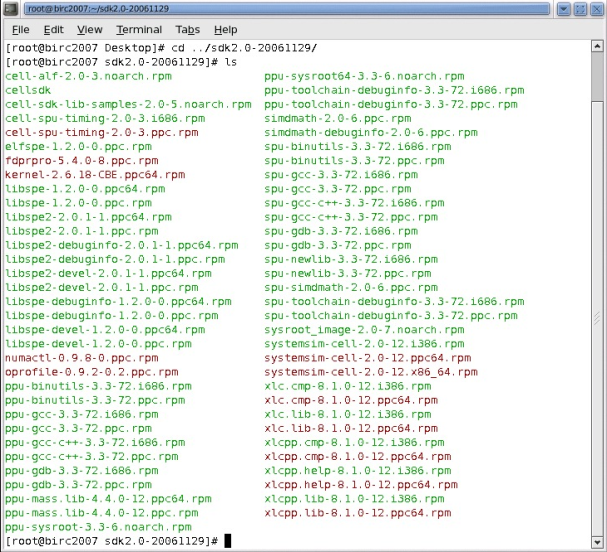


1. List of rpm packages for Cell SDK v2.0
2. To set the permission of these rpm packages to “executable”, open Terminal and type “chmod +x *.rpm”

*Install Cell SDK v2.0:*

1. There is no need to install Cell System Simulator, as there is a real Cell processor on the PS3 machine. Hence, under Terminal, *cd* to the folder containing rpm packages for Cell SDK v2.0, type “./cellsdk install --nosim”
2. Type “yes” when prompted by the installer
3. Upon successful installation, type “./cellsdk verify” and hit ENTER. The display should be similar to Figure 4.


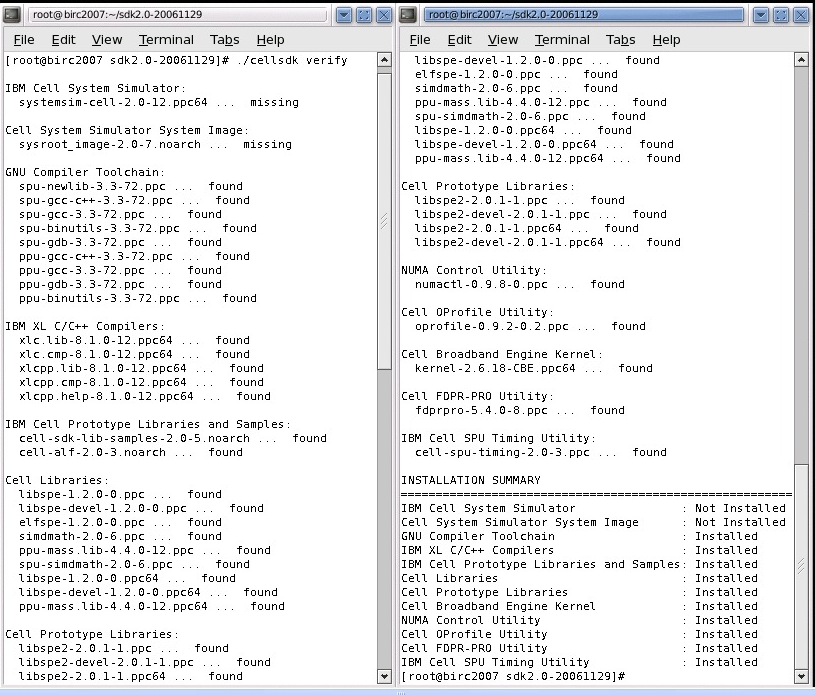


1. Post-installation verification

*If there is error during installation:*

1. Remove all installed Cell SDK rpm packages
2. Perform manual installation of the rpm package using “Software installer”, following the order shown in Figure 4. If any rpm package appears to be corrupted, re-download the file by web browser or “yum”
3. Upon successful installation, type “./cellsdk verify” and hit ENTER. Again, the display should be similar to Figure 4.

*Test by running a program on Cell processor:*

1. Now it is ready to build and run a program on the PS3 machine. Try any sample Cell BE code, such as *spe_distance.c* and *ppe_distance.c* from <http://www.ibm.com/developerworks/power/library/pa-linuxps3-1/>
2. Open a terminal and type the following:

| #Compile the SPE program  spu-gcc spe_distance.c -o spe_distance  #Embed the SPE program into an ELF object file, and expose it  #through the global variable: calculate_distance_handle  embedspu calculate_distance_handle spe_distance spe_distance_csf.o  #Compile the PPE program together with the SPE program  gcc ppe_distance.c spe_distance_csf.o -lspe -o distance  #Run the program  ./distance |
| --- |

1. If this works fine, the PS3 has been properly configured. In summary, installing Linux on the PS3 takes a little bit of effort, but in the end you get a low-cost, fully working Cell Broadband Engine processor.

## 1.2 Workstation setup

### 1.2.1 Dual boot for MS Windows XP and Fedora Core 5

Fedora Core 5 is the only recommended operating system for Cell SDK v2.0 by IBM. Being one of the fastest growing operating system in which a new version is released every three months, Fedora provides one of the most reliable platforms for developing high-performance application.

With the lack of physical hardware, it can be desirable to use dual-OS mechanism on the same machine. This section will show the detailed instruction of install Fedora Core 5 as a second operating system on a machine currently running Microsoft ® Windows XP.

*Preparation*

1. Download the ISO disk image for Fedora Core 5 from <ftp://download.fedora.redhat.com/pub/fedora/linux/core/5/i386/iso> and burn into the respective CDs or DVDs
2. Download the ISO disk image for GParted Live CD from <http://gparted.sourceforge.net/download.php> and burn into the respective CD

*Disk partitioning*

1. Reboot the PC, set the CD/DVD Drive as the first boot device
2. Insert GParted Live CD, the system will boot into GParted as shown in Figure 6

[
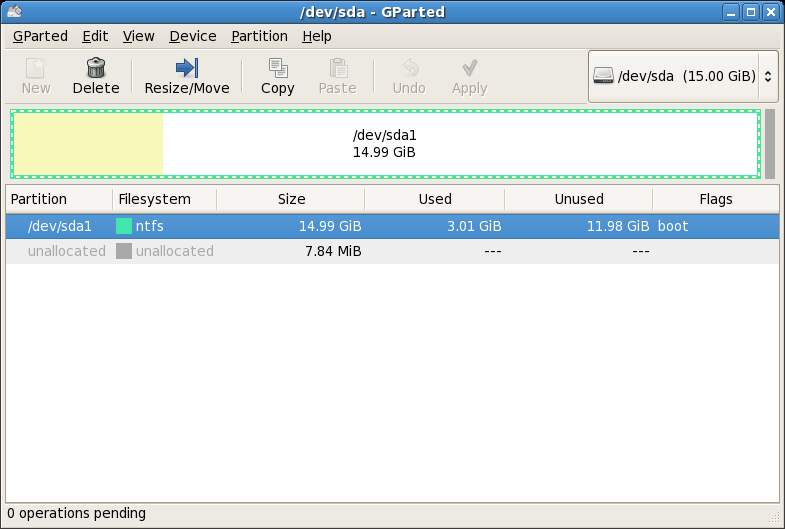
](http://www.techotopia.com/index.php/Image:Fedora_linux_gparted_main.jpg)

1. GParted user graphical interface
2. Select the NTFS partition in the GParted window and click on the Resize/Move toolbar button to invoke the Resize/Move dialog, as in Figure 7

[
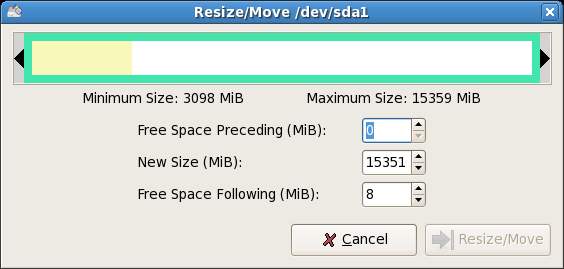
](http://www.techotopia.com/index.php/Image:Fedora_gparted_resize.jpg)

1. Resize/Move dialog
2. Change the New Size field to 8000 MB and click on the Resize/Move button at the bottom of the dialog. The Resize action will then appear in the Pending panel at the bottom of the main GParted screen and the new space will be displayed in the graphical representation of the disk as Unallocated space, as in Figure 8

[
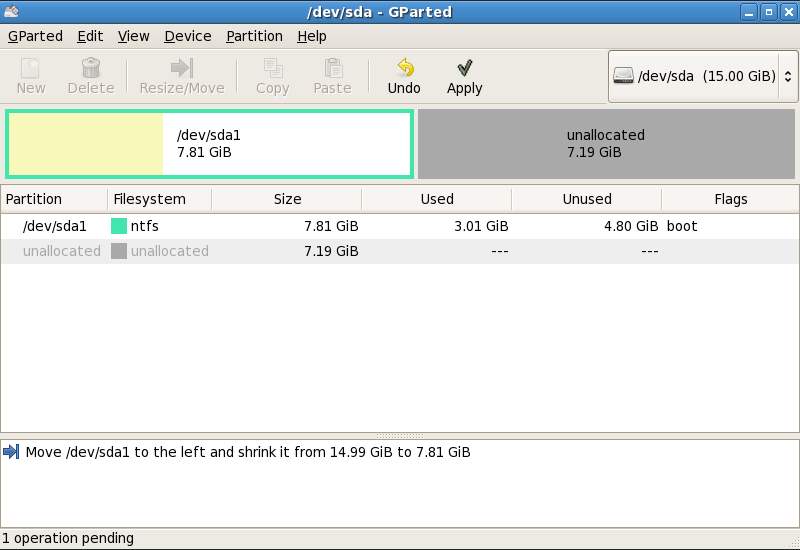
](http://www.techotopia.com/index.php/Image:Fedora_gparted_resize_pending.jpg)

1. GParted after resizing
2. Click “Apply” to perform disk partitioning. The disk is now partitioned with the existing Windows installation and unallocated space suitable for the installation of Fedora Linux.

*Fedora Core 5 installation*

1. Reboot the computer, Fedora CD/DVD to boot
2. On the partitioning screen, select the “Create custom layout” and choose the unallocated space resulted from the disk partitioning
3. Follow the instruction on the screen until the new operating system is successfully installed

It should be noted that depending on the hardware, certain incompatibility problems may occur. These issues will be addressed in Chapter 4.

### 1.2.2 Cell SDK v2.0

The installation of Cell SDK on the Workstation is similar to that on the Cell Box. However, the Workstation should have System Cell Simulator install. To perform this, use the command “./cellsdk install” instead of “./cellsdk install --nosim”.

It is crucial that the configuration and rpm packages installed are the same for Workstation and Cell Box. The reason is that the program will be build on the Workstation, uploaded to the Cell Box and executed on the Cell Box, as shown in Figure 9. This will complement the limitation of the Cell Box having memory size of only 256MB. On the other hand, the Workstation has all required configurations for Cell application development as specified by IBM, which is Pentium IV and memory size of 1GB and above.

1. Diagram of Cell application development

The test to verify that Cell SDK v2.0 is successfully installed is delayed until the IDE has been installed.

### 1.2.3 IDE for Cell application development

To create a Cell BE-specific project environment, Eclipse 3.2, CDT 3.1.2, and Eclipse IDE plug-ins for Cell BE (CellDt) need to be installed.

*Install Sun Java 1.4.2*

1. Download JRE v.1.4.2 rpm package from <http://java.sun.com/j2se/1.4.2/download.html> . The default Java VM (GCJ) that comes installed with Fedora Core 5 is not sufficient. Install the rpm package by Package Manager
2. Make sure that your JAVA_HOME and your PATH are updated accordingly. For example: JAVA_HOME=/usr/java/j2sdk1.4.2_13/jre and PATH=$PATH:$JAVA_HOME/bin
3. “java –version” should show the following

java version "1.4.2"

gij (GNU libgcj) version 4.1.0 20060304 (Red Hat 4.1.0-3)

Copyright (C) 2006 Free Software Foundation, Inc. This is free software; see the source for copying conditions. There is NO warranty; not even for MERCHANTABILITY or FITNESS FOR A PARTICULAR PURPOSE.

*Install Eclipse 3.2*

1. Download 3 packages: eclipse-SDK-3.2-linux-gtk.tar.gz, org.eclipse.cdt-3.1.0-linux.x86.tar.gz and com.ibm.celldt.update.tar.gz into /opt/IDE
2. Install Eclipse 3.2 by “tar xvfz eclipse-SDK-3.2-linux-gtk.tar.gz”
3. Install CDT 3.1 plug-ins by “tar xvfz org.eclipse.cdt-3.1.0-linux.x86.tar.gz"
4. In Eclipse, click Help => Software Updates => Find and Install...
5. Select Search for new features to install, click Next.
6. Click New Local Site...
7. Go to /opt/cellsdk/IDE/com.ibm.celldt.update, and click OK.
8. Click Finish and follow the on-screen instructions.

## 1.3 Build and run applications with Eclipse IDE

This section will provide a step-by-step guide from creating a CBEA project until debugging in Eclipse IDE.

*Initialize Eclipse*

1. Start Eclipse
2. Open the C/C++ perspective by selecting Window > Open Perspective > Other....
3. Choose C/C++ from the list and click OK.

*Create SPU project in Eclipse*

1. Create a new C project by clicking File > New > Project.
2. In New Project wizard: expand the section C and select Managed Make C Project. Click Next.
3. The project needs a unique name, such as SPU. Click Next.
4. For Project Type, select Cell SPU Executable, and then click Next.
5. Click Finish.
6. In the C/C++ Projects view, right click on the SPU project, and select Properties.
7. Click on C/C++ Build in the left pane, then under SPU GNU C Compiler with Debug Options, select Directories. In the Include Paths pane on the right, press Add.
8. Type “/opt/ibm/cell-sdk/prototype/src/include/spu”, then return to the C/C++ Perspective by pressing OK twice.
9. Create a .c source file by clicking File > New > Source File. Specify the name, e.g. spu.c
10. Project build: Save the source file (Control + S). The project is built automatically. The build output is displayed in the Console view, and new files, such as “binaries” and “includes”, are shown in the C/C++ Projects view.

*Create PPU project in Eclipse*

1. Click File > New > Managed Make C Project
2. Specify the project name, such as *PPU*. Click Next
3. For Project Type, select Cell PPU Executable. Click Next.
4. In Project Preference tab, check the SPU project that is used in the PPU project, such as *SPU.* Click “Finish”
5. In the C/C++ Projects view, right click on the PPU project, and select “Properties”
6. In the left pane, select C/C++ Build.
7. In the Tool Settings tab, click on Libraries (under the PPU GNU 32 bit C Linker category), then click “Add” in the “Libraries” pane on the right. Type “spe” in the dialog box
8. In the Tool Settings tab (under PPU GNU 32 bit Embed SPU), select the Inputs option, and then click Add in the Embed SPU Input pane. Click “Workspace” button and point to the SPU binary file, such as *SPU*
9. To create a PPU source file: Right click on the PPU project, and select File > New > Source File. Specify the name, such as ppu.c
10. Similar the SPU project, the project is built automatically each time “Save” action is made

*Run and debug the program from a remote Cell Box*

1. In the Cell Environments view at the bottom, right click on Remote Cell Box and select Create
2. Specify the Target name, such as PS3. Also, the IP address and login information of the remote Cell Box need to be specified. Click “Finish”
3. Select the PS3 environment, and click on Start the Environment (green arrow).
4. Click Run > Debug. In the left pane, right click on C/C++ Cell Target Application, and select New. In the Main tab of the debug configuration, ensure that project PPU is in the Project field
5. Navigate to the Target Selection tab. For the Choose Target field, select PS3 (the Cell Box environment)
6. In Synchronize tab, specify the files to be uploaded to the remote Cell Box. Error may occur if there is no files uploaded
7. In Debugger tab, to debug both PPU and SPU projects at the same time, select “Cell BE gdbserver”. Uncheck “Stop on startup at: main”
8. Click Debug to launch the debug session.
